# The Role of Nursing Staff Regarding Goal Setting and Achieving in Geriatric Rehabilitation: A Focus Group Study

**DOI:** 10.1097/RNJ.0000000000000429

**Published:** 2023-08-15

**Authors:** Anne Marie Vaalburg, Elizabeth M. Wattel, Petra Boersma, Cees M. P. M. Hertogh, Robbert J. J. Gobbens

**Affiliations:** 1 Department of General Practice and Elderly Care Medicine, Amsterdam Public Health Research Institute, Amsterdam UMC, Vrije Universiteit Amsterdam, Amsterdam, the Netherlands.; 2 Faculty of Health, Sports and Social Work, Inholland University of Applied Sciences, Amsterdam, the Netherlands.; 3 University Network of Organizations for Care for the Elderly of VU University Medical Centre, Amsterdam UMC, Vrije Universiteit Amsterdam, Amsterdam, the Netherlands.; 4 Ben Sajet Center for Long-Term Care, Amsterdam, the Netherlands.; 5 Zonnehuisgroep Amstelland, Amstelveen, the Netherlands.; 6 Department of Primary and Interdisciplinary Care, Faculty of Medicine and Health Sciences, University of Antwerp, Antwerp, Belgium.; 7 Tranzo, Tilburg School of Social and Behavioral Sciences, Tilburg University, Tilburg, the Netherlands.

**Keywords:** Nurses’ role, geriatric nursing, rehabilitation, goal setting

## Abstract

**Purpose:**

The aim of this study was to explore and clarify the role of nursing staff in geriatric rehabilitation on supporting patients in goal setting and achieving, through reflecting on rehabilitation interventions.

**Design:**

A descriptive qualitative study was conducted.

**Methods:**

We conducted four online focus group interviews with 23 members of the nursing staff working in geriatric rehabilitation. They reflected on six interventions, preclassified into three types: setting goals in the admission phase, increasing patient participation in order to personalize the rehabilitation trajectory, and supporting patients in working on short-term goals. Data were analyzed using thematic content analysis.

**Results:**

Setting goals in the admission phase is primarily the task of the multidisciplinary team rather than the nursing staff. Interventions to increase patient participation align with the coordinating role of nursing staff in the rehabilitation team. Working on short-term goals is of great value to patients.

**Clinical Relevance to the Practice of Rehabilitation Nursing:**

The connection between the patient’s personal goals and professional treatment aimed at functional recovery can be enhanced by strengthening the position of nursing staff working in geriatric rehabilitation.

**Conclusion:**

Members of nursing staff in geriatric rehabilitation see themselves playing a coordinating role in the multidisciplinary team, supporting the patient in goal work. Interventions aimed at advancing patient participation and providing support for short-term goals reinforce this role.

## Introduction

Older people can recuperate in special rehabilitation wards in nursing homes following an acute or subacute decrease in function after a medical event such as a hip fracture or stroke (Van Balen et al., 2019). In 2019, 53,320 patients in the Netherlands were admitted to a so-called “geriatric rehabilitation facility,” their average length of stay being 42 days (Vektis, 2021). Van Balen et al. (2019) describe two core principles of geriatric rehabilitation: working with an interdisciplinary approach in a multidisciplinary team and a structured and time-delimited rehabilitation plan focused on the goals of the patient. The multidisciplinary team includes an elderly care physician, a physiotherapist, a nurse, and an occupational therapist (Holstege et al., 2017), and according to the patients’ needs, the team can be extended to include a speech therapist, a dietician, a psychologist, and a social worker (Van Balen et al., 2019).

In Dutch healthcare facilities for older people, geriatric rehabilitation is relatively new. In 2015, the healthcare system was changed from a long-term care government-guided reimbursement system without financial incentive for “efficient” geriatric rehabilitation toward a more market-guided bundled payment system (Holstege et al., 2015). This change necessitated several activities aimed at professionalizing geriatric rehabilitation. For example, appropriate guidelines and care pathways have been developed for stroke and fractures (Holstege et al., 2017). In addition, Tijsen et al. (2023a, 2023b) strived to identify and formalize factors that contribute to a challenging rehabilitation environment to optimize rehabilitation care.

Geriatric rehabilitation mainly takes place in nursing homes. Nursing staff in nursing homes in the Netherlands consist of registered nurses, but mostly certified nursing assistants (Rommets & Roelvink, 2022). Compared to other countries, the Dutch-certified nursing assistant education is rather lengthy, specifically consisting of a 3-year practice-oriented course (van Wieringen et al., 2022). Nursing staff were traditionally more focused on providing comfort in an environment where older people live, as opposed to working within a multidisciplinary team with patients on rehabilitation goals needed to safely return to home. Nursing home managers conclude that there are major differences between long-term care and geriatric rehabilitation with different demands on the professionals involved (Jongenburger, 2019). An increase in the number of registered nurses working in geriatric rehabilitation has been seen (Inspectie Gezondheidszorg en Jeugd, 2022). One of the specific aspects that the nursing staff have been struggling with was voiced by De Vos et al. (2018). DeVos et al. found that nursing staff in geriatric rehabilitation in the Netherlands experience a certain apprehensiveness about performing goal-centered care in multidisciplinary teams. Clear working procedures might help the nursing staff in taking on their role (Cameron et al., 2018; De Vos et al., 2018; Loft et al., 2017).

Vaalburg et al. (2023) performed a scoping review to explore the range of interventions on goal setting and achieving available to nurses in geriatric rehabilitation. The researchers found 11 interventions, of which six provide a sufficiently clear description of the nursing role in an inpatient rehabilitation setting (see Table 1). These six interventions can be categorized into three types. The first type of interventions focuses on goal setting with the patient in the admission phase with the aid of a scale that measures the degree of independence in certain functions (Lorensen’s Self-Care Capability Scale [Ruland et al., 1997] and the Collaborative Functional Goal Setting [Smit et al., 2018]). The second type of interventions focuses on increasing patient participation to personalize the rehabilitation trajectory using the Motivational Interviewing Intervention (Gual et al., 2020) or the Increased Participation Intervention (Holliday et al., 2007; Van De Weyer et al., 2010). The third type of interventions is meant to provide support for patients as they work on short-term goals with an exercise book or goals written on a whiteboard (map with prescribed exercises [Huijben-Schoenmakers et al., 2013] based on the Dutch Stroke Guidelines [Hafsteindottir & Schuurmans, 2009] or the Collaborative Patient Goal-Setting Initiative [Revello & Fields, 2015]). It is unclear to what extent these six interventions, or aspects of these interventions, align with the professional view of nursing staff in geriatric rehabilitation in the Netherlands on the role they could play in the multidisciplinary team with patients’ goal setting and achieving. With the aim to further explore and clarify this role, a focus group study was designed with geriatric rehabilitation nurses. The six interventions, as well as their underlying vision and elaboration in activities, were used as a means to reflect on this role.

**Table 1:** Interventions

Intervention Type 1, 2, or 3	Authors (Year) Country Name of Intervention	Aim of Intervention	Intervention Description	Accompanying Materials
Type 1 Interventions focused on goal setting with the patient in the admission phase using a scale	Ruland et al. (1997) United States Lorensen’s Self-Care Capability Scale (refined)	Tailoring nursing care decisions to desired outcomes as preferred by individual patients.	1. The patient is asked to name predominant problems and to indicate which are most important to relieve. 2. The nurse and the patient assess performance on each self-care function with the help of Lorensen’s Self-Care Capability Scale. 3. Together, they select the desired level of functioning and evaluate the progress.	The Lorensen’s Self-Care Capability Scale is composed of 13 dimensions of patient’s self-care abilities.
	Smit et al. (2018) The Netherlands Collaborative Functional Goal Setting	Facilitate the process of jointly setting goals by the use of a measurement instrument.	1. On admission, the nurse completes the Barthel Index or functional items of the Utrecht Scale for Evaluation of Rehabilitation. Scale scores are presented in a multidisciplinary meeting (MDM). 2. The multidisciplinary team sets functional goals. 3. Goal-setting meeting with the patient and the physician/nurse practitioner. The patient is invited to set their own functional goals. The patient’s goals are defined through shared decision-making between the patient and the physician. 4. Prior to biweekly MDMs, a new functional assessment is conducted by nurse. 5. During MDM, the target scores are reviewed. 6. The physician/nurse practitioner informs the patient about the outcome of MDM.	Two standardized functional measurement instruments: the Barthel Index and the Utrecht Scale for Evaluation of Rehabilitation.
Type 2 Interventions focused on increasing patient participation in order to personalize the rehabilitation trajectory	Gual et al. (2020) Spain Motivational Interviewing Intervention	To empower, motivate, and engage the person in planning and participating in the rehabilitation plan in order to improve rehabilitation outcomes, increase person satisfaction and self-efficacy.	Four sessions of motivational interviewing by nurses: 1. Engage the stroke survivor in their care. 2. Co-create a personalized rehabilitation plan, complementing the routine geriatric rehabilitation plan. 3. Reinforce engagement and adherence to the plan to maintain behavior change and functional improvement.	Personalized rehabilitation plan agreed between stroke survivors and nurses based on stroke survivor’s goals, needs, preferences, and capabilities.
	Holliday et al. (2007) United Kingdom Van De Weyer et al. (2010) United Kingdom Increased Participation Intervention	Involving patients in goal setting in order to self-manage, maintain independence, and achieve the best possible quality of life.	Prior to admission, the patient is given a workbook that explains the goal-setting process in detail. On the day of admission, the key worker (assigned to every patient) interview focuses on the patient experience to facilitate advocate role within goal setting. On the week of admission, the key worker works with the patient to complete the workbook. On the Friday of admission, week’s goals set by therapists and the patient working together. The patient is present in goal-setting meetings. Short-term goals are reset on two or three weekly cycles.	Patient workbook: 1. Prioritize activity and participation domains. 2. Identify specific tasks within those domains patient wishes to work on. 3. Determine rehabilitation goals achievable within time frame of admission.
Type 3 Interventions are meant to support patients in working on daily goals	Huijben-Schoenmakers et al. (2013) The Netherlands Map with prescribed exercises based on Stroke Guidelines	Increase practice time of patients through nursing involvement.	Each week, exercises based on interventions from the Clinical Nursing Rehabilitation Stroke Guidelines are adapted to individual goals and rehabilitation level of the patient.	4. Exercises are documented in exercise map.
	Revello & Fields (2015) United States The Collaborative Patient Goal-Setting Initiative	Better patient outcomes through nurse and patient collaborative goal setting.	1. Each day, the nurse supports the patient setting 1–2 goals they hope to achieve in the following 24-hour period. 2. The following evening, goal achievement is evaluated, and the patient either continues the previous goals or names new ones. 3. Nurses on other shifts and therapists acknowledge the goals with the patient and make an effort to see that the goals are met.	Whiteboards in patients’ rooms.

## Methods

This descriptive qualitative design study used focus group interviews with nursing staff working in geriatric rehabilitation to explore perspectives on their role in working with patients on their rehabilitation goals. The study followed the 32-item Consolidated Criteria for Reporting Qualitative Research formulated by Tong et al. (2007).

### Sample

Participants were recruited through a purposive sampling strategy. Supplying potential participants with an invitation leaflet with information about the purpose and procedure of the focus groups, we contacted nursing home managers and senior staff members of two research networks (UKON and UNO Amsterdam) and a quality network (GRZ E-cademy). The managers and senior staff were asked to approach “outstanding” members of their nursing staff and encourage them to volunteer for the focus groups. “Outstanding” was described as “visibly concerned with their profession and preferably in the possession of a rehabilitation nursing diploma or similar qualification.” The other inclusion criterion was working in inpatient geriatric rehabilitation. To the participants who volunteered, four different dates for focus groups were provided. Participants chose a group based on their schedule. Participants received a €10 voucher for cosmetics.

### Information and Interview Guide

A semistructured interview guide was developed by all authors (AMV, EW, PB, CH, RG). The guide consisted of three questions: (1) Does this intervention/part of the intervention meet the needs of patients in your practice? (2) Does this intervention/part of the intervention fit the role of nursing staff? (3) Which preconditions are needed to use this intervention in practice? The interview guide was tested in a pilot session with one nurse and three nursing students on a geriatric rehabilitation ward. No revisions were suggested to the guide; however, some revisions were made to the PowerPoint that was used to present the six interventions.

### Procedure

Four online focus group interviews were conducted in April and May 2022. All focus group interviews were moderated by one researcher (AMV); PB took notes to provide feedback to the moderator. After introducing the six interventions, divided into three types, participants were asked to reflect on the three types by initially writing down their thoughts on a digital whiteboard. This step provided each participant with an equal opportunity to contribute to the quest for which these focus groups were designed. The written information helped the moderator select topics and invite quieter participants to clarify their notes. Each focus group had five to seven participants. The interviews lasted about 120 minutes, were audio-recorded, and were transcribed verbatim.

### Analysis

Data were analyzed using thematic content analysis (Green & Thorogood, 2018), which aims to provide a “map” of the content of the data set and an overview of variation and regularities within the data. To maintain rigor, analyses were independently performed by two researchers (AMV, PB) (Johnson et al., 2020). The analysis started with thoroughly reading the transcripts and the written comments on the digital whiteboard to become familiar with the data. Then, the transcripts and written comments were read sentence by sentence, and quotes that appeared to answer one of the three key questions were included in a matrix set up for the analysis. Subsequently, the assembled quotes were read, and words that appeared to articulate key thoughts were highlighted. Based on the highlighted fragments, notes were made to catch the first impressions, thoughts, and initial analysis. From these notes, a coding tree was developed by the two of the researchers (AMV, PB). With the other authors (EW, CH, RG), the interim analyses were discussed, while constantly reflecting on potential author bias.

### Ethics

This study was not subject to the Dutch Medical Research Involving Human Subjects Act; therefore, it did not undergo a review by a medical ethics committee (Central Committee on Research Involving Human Subjects, n.d.). After being given information about the study, the participants gave consent for the audio recording of the interviews and for their personal data to be retained. No family names or other person-level information that can be traced back to individuals were used in the transcriptions.

## Results

A total of 23 members of nursing staff working on geriatric rehabilitation wards participated. Table 2 gives an overview of the participants’ general characteristics. Table 3 summarizes the results of the study.

**Table 2:** Characteristics of the Focus Group Participants

Participant	Focus Group	Age (years)	Level of Basic Nursing Education	Advanced Training in Rehabilitation^a^	Working Years in Geriatric Rehabilitation
1	1	53	EQF4	+	15
2	1	49	EQF6	−	17
3	1	39	EQF4	−	8
4	1	52	EQF4	+	7
5	1	56	EQF4	+	11
6	2	28	EQF4	+	8
7	2	26	EQF4	+	6
8	2	41	EQF6	+	11
9	2	26	EQF6	−	1
10	2	30	EQF4	−	5
11	2	46	EQF3	+	22
12	3	42	EQF4	−	2
13	3	39	EQF4	−	1
14	3	37	EQF6	+	13
15	3	52	EQF4	+	9
16	3	50	EQF4	+	4
17	3	53	EQF3	−	4
18	3	60	EQF4	+	4
19	4	27	EQF6	+	5
20	4	47	EQF6	−	6
21	4	26	EQF4	+	4
22	4	26	EQF6	−	7
23	4	36	EQF4	−	10
		Mean = 40.9	2 (EQF3) 14 (EQF4) 7 (EQF6)	+57%	Mean = 7.8

*Note.* EQF = European Qualifications Framework—describes levels of qualification ranging from basic (Level 1) to advanced (Level 8); EQF3 = certified nursing assistant—completed 3 years of practice-oriented nursing education in a regional education center for vocational training; EQF4 = vocational nurse—completed 3 .5– 4 years of nursing education in a regional educational center for vocational training and registered in the Dutch Register for care professionals (so-called BIG register); EQF6 = nurse with bachelor’s degree obtained from a university of applied sciences and registered in the Dutch Register for care professionals.

^a^ Continuing education in geriatric rehabilitation for both nurses and certified nursing assistants exists and often takes place on the initiative of the healthcare institutions where they are employed.

**Table 3:** Results

	1. Do (Part of) These Interventions Meet the Needs of Patients in Your Practice?	2. Do (Part of) These Interventions Fit the Role of the Nurse?	3. Which Preconditions Are Needed to Use (Part of) These Intervention(s) in Practice?
Intervention Type 1 Collaboratively setting goals in the admission phase with the aid of a measurement instrument	Collaboratively setting goals promotes patient engagement. Participation by patients in the goal-setting process is hindered, mainly by cognitive impairment. Patients need to be helped in providing optimal input.	Setting goals is usually done by the multidisciplinary team, not by the nurse. The nursing role in this phase is coordinating. The role of the nurse is to assess the extent to which control should be taken over from the patient in the admission phase. Cooperating with family is an important nursing task. In the admission phase, certain nursing focus points are missed.	Measures have to be taken to make participating in collaborative goal setting possible for the patient. To work with goal setting instruments in the admission phase, training is needed of which explanation of the added value of the instrument is part.
Intervention Type 2 Increasing patient participation during the rehabilitation process	Multidisciplinary team meetings are appropriate moments to involve patients. Patients need to be helped in providing optimal input.	Supporting the patients to participate in order to make the rehabilitation process as person-centered as possible is a central feature of the nurses’ work. The nurse connects in multiple ways: -by translating jargon, -by linking professional goals and patients’ lives, -by explaining that every activity can be seen as rehabilitation, etc.	Being able to have motivational conversations is a key skill. Nurses should work consistently with rehabilitation plans. Working with integrated goals instead of discipline-specific goals supports the coordinating role of the nurse. Not feeling in the position to take on a coordinating role in the multidisciplinary team.
Intervention Type 3 Supporting patients in working on short term goals	Clear short-term goals have many benefits for the patient: -short-term goals make rehabilitation manageable, -achieving small goals motivates, -patients do not have to wait for the next therapy session to exercise, -small goals help families take on supportive role, -small goals support interprofessional collaboration and provide a consistent team approach, which in turn is clarifying for the patient.	Management of these interventions is the responsibility of other disciplines. The nurses’ role is to monitor progress and inform other disciplines about progress. Differing opinions about whether stimulating the patient to exercise is the nurses’ task: “extra” workload versus most exercises fit in daily activities. Interventions are a means of involving family, which is seen as an important nursing task.	Exercise sheets and whiteboard with short-term exercises need to be kept up-to-date to ensure continuity.
Preconditions for all three types of interventions	Time to apply the interventions. Care pathways and work procedures describing the nurse’s and the patient’s role securing the patient’s participation and the specific nursing contribution. Education in geriatric rehabilitation. Nursing leadership.		

### Interventions Type 1: Collaboratively Setting Goals in the Admission Phase

The first two interventions use scales to set goals with the patient in the admission phase (see Table 1), specifically the Barthel Index (Mahoney & Barthel, 1965), the Utrecht Scale for Evaluation of Rehabilitation (Post et al., 2009), or Lorensen’s Self-Care Capability Scale (Ruland et al., 1997). The different scales are composed of items focusing on self-care abilities and mobility.

#### Patients’ Needs

Participants saw the value of these interventions for patients. Collaboratively setting goals promotes patient engagement.

…if you use the Lorensen’s Scale, you make the patient think, “Oh, what are my problem areas?” And by showing them what the problem areas are, you can also include them in the goals and I think they will be more encouraged to actually achieve those goals because they were involved in setting the goals. (Participant 9)

At the same time, participants identified factors that hinder the geriatric patient from actively participating in the goal-setting process, the main factor being cognitive impairment and its consequences. Patients are not always able to oversee the situation, to plan, or to retain information.

#### Role of Nursing Staff

Setting goals is usually done by the multidisciplinary team, with each discipline focusing on their own field of expertise, and not by the nursing staff. When it comes to goal setting during the admission phase, participants describe their role as coordinating:

I think as a nurse you are, especially in the multidisciplinary team, a cog in the wheel, you’re there most of the time. We do the admission and we check all kinds of things, and in the case of pressure sores, we inform the occupational therapist: please come and see the patient today, and if the patient does not need a wheelchair, she can come a day or two later. We try to coordinate, to direct…. (Participant 16)

The role of nursing staff is to assess the extent to which the multidisciplinary team should take over control from the patient in the admission phase and to decide whether temporary goals need to be set that are checked with the patient at a later stage and whether, in this stage, the family should be questioned about goals. The family supplies the multidisciplinary team with essential information about the patient and their needs. Concurrently, they, like the patient, need to be informed about rehabilitation and its possibilities and limitations. Cooperating with family was seen by the participants as an important task of nursing staff.

Reflecting on these interventions, participants made clear that, in the admission phase, certain nursing focus points are regularly missed, such as wound care and medication management, because they do not belong to the expertise of the other rehabilitation professionals.

…within the geriatric rehabilitation there’s a lot of goal setting from the disciplines. It’s big, walking, the occupational therapist who’s busy cooking, for example. But a care team, yes, the catheters, all the nursing supplies, […]teaching someone to go home with the catheter and emptying it. Those are all goals that are often, yes, forgotten. (Participant 3)

#### Terms and Conditions for Implementation of Interventions Type 1

Participants considered collaboratively setting goals with a scale the responsibility of the multidisciplinary team as a whole and not as a specific task of nursing staff. They did, however, have ideas about measures to facilitate this collaborative setting of goals with the patient, for example, a multidisciplinary intake to avoid overlapping questions and thus unnecessarily burdening the patient, giving the patient time to acclimatize and unwind after traveling from the hospital, and postponing the goal setting till the second or third day after admittance. Whoever on the multidisciplinary team uses the instruments, such as the Utrecht Scale for Evaluation of the Rehabilitation, the Barthel Index, and Lorensen’s Self-Care Capability Scale, should be trained. Attention should be paid to the added value of using such an instrument.

#### Interventions Type 2: Increasing Patient Participation During the Rehabilitation Process

The second two interventions focus on increasing patient participation in order to personalize the rehabilitation trajectory (see Table 1).

#### Patients’ Need

Participants see the multidisciplinary team meetings as appropriate moments to encourage patient involvement, as is done in the Increased Participation Intervention (Holliday et al., 2007; Van De Weyer et al., 2010). Similar to the admission phase, participants stated that, during rehabilitation, patients need to be supported to be optimally involved. One of the participants gave an example of how this is done on her ward.

In preparation of the multidisciplinary team meeting, we give the patient a short questionnaire. We have been doing this for two weeks now, because we would like to include more person-centered elements and know: “what are the patient’s goals and are they realistic?” […]. These two weeks we’ve received 10 completed forms, and it’s really good to see that patients come up with: “I’m scared.” Something that has not come up in daily practice. (Participant 22)

This questionnaire has the same goal as the sessions in the Motivational Interviewing Intervention: “active listening to persons’ concern and adaptation to the rehabilitation plan” (Gual et al., 2020, p. 5).

#### Role of Nursing Staff

Participants’ practices do not include patient attendance at the multidisciplinary team meetings, as is part of the Increased Participation Intervention, or a set number of planned meetings between nursing staff and the patient, as is done in Gual et al.’s (2020) Motivational Interviewing Intervention. However, supporting the patients to participate to make the rehabilitation process as person-centered as possible is seen as a central feature of the work of nursing staff and is ingrained in their practice because of their 24/7 presence on the ward.

Your contact with the rehabilitant, being first point of contact, help the rehabilitant by explaining the rehabilitation in understandable language and motivate them to rehabilitate. Looking for the intrinsic motivation… (Digital whiteboard comment)

The role of nursing staff was described as “helicopter,” “connector,” and “coordinator” on several levels for example as an interpreter of jargon.

We are the link between the patient and the rest. Because they all talk medically, and they all have goals, and the patient is sitting there flapping his/her ears. And you are the one who has to say: If this is it, then we are going to do it like this…. (Participant 4)

Another explanation of the concept of connector is the connecting link between professional goals and patients’ lives:

With the helicopter view, I mean to say: What’s in it for the rehabilitant? What’s important for them? […] we once had someone from the country, […] he had to learn to walk, but this man just wanted to sit in his chair and pull potatoes out of the ground. And not the walking. (Participant 2)

The premise that “everything is rehabilitation” needs to be explained to patients. This is a third interpretation of the connecting or more precise, integrating role.

They see going to the physiotherapist as rehabilitation, unlike washing and getting dressed, and we are responsible to make them understand: How will you manage in the future? (Participant 8).

In addition, the role of nursing staff was defined as “motivator” or “driving force” for the patient, and virtually, all participants mentioned the importance of being able to have motivational conversations as a key skill.

And as for Intervention 4 [Motivational Interviewing Intervention, AMV]…you do this constantly. From the conversations you hear a lot and often find out why a client is struggling with something or why things don't work out. (Digital whiteboard comment)

#### Terms and Conditions for Implementation of Interventions Type 2

Education in motivational interviewing was emphasized by participants as a prerequisite to help members of the nursing staff make the transition from caring for patients to coaching them to work on their personal goals. Another prerequisite is that nursing staff should consciously work with plans; otherwise, they will not be able to support the patient in participating in the rehabilitation process and play a coordinating role.

I think half the team is not aware of treatment plans. (Participant 10)

Nursing staff have the potential to play a coordinating role, being closest to the patient and always in a position to talk about and observe their progress. Participants named several factors that stand in the way of the coordinating role, such as working with discipline-specific goals and the lack of an integrated rehabilitation plan.

…it is very much discipline-oriented. In the treatment plan the occupational therapist makes a goal, the physiotherapist makes a goal, […]. And if it’s about walking […] the nurse automatically is of less importance. (Participant 3)

Also, not all nurses feel in a position to play a coordinating role in the rehabilitation process, as illustrated by the last quote.

The therapist focuses on the therapy, but I expect the nurses to observe: “This patient has had a couple of down days. I do not think it realistic to send him home yet.” […] But we do not always see a possibility to utter this…. (Participant 8)

### Interventions Type 3: Supporting the Patient to Work on Short-Term Goals

Finally, two interventions meant to support patients in working on short-term goals were discussed with the focus group participants (see Table 1).

#### Patients’ Needs

Participants saw many advantages for the patient in working with interventions that support working on short-term goals, such as exercise goals on whiteboards or exercise sheets. Short-term goals make rehabilitation manageable, achieving small goals motivates, patients do not have to wait for the next therapy session to exercise, and it helps families take on a supportive role. Ultimately, these interventions support interprofessional collaboration on goals and thus provide a consistent team approach, which in turn is clarifying for the patient.

I think the exercise sheet is also a nice method, because the rehabber has exercises at any time of the day/week and is not “waiting” for the therapy moments of the physio/ergo/etc. (Digital whiteboard comment)

#### Role of Nursing Staff

In general, the initiative and management of these interventions, such as exercise goals on whiteboards or exercise sheets, were considered the responsibility of other disciplines. The role of nursing staff is to monitor progress and inform other disciplines about progress. Participants had differing opinions about whether stimulating the patient to exercise is the nurses’ task. Some see this as “extra” workload; others emphasized that if you work according to the philosophy that “everything is rehabilitation,” most exercises fit into daily activities. Involving family in the rehabilitation process was seen as an important nursing task.

#### Terms and Conditions for Implementation of Interventions Type 3

Exercise sheets and whiteboard with short-term exercises need to be kept up-to-date to ensure continuity.

### Terms and Conditions for Implementation of All Three Types of Interventions

Some of the conditions for implementation mentioned by the participants apply to all interventions. First, time is needed to actually apply the different interventions, and second, care pathways and work procedures describing the role of nursing staff and the patient’s role secure the patient’s participation. It legitimizes certain activities, such as patient attendance at the multidisciplinary team meeting or having motivational interviewing sessions, and makes these activities less team dependent. Third, the need for education in geriatric rehabilitation was mentioned in all focus group interviews. Educating nursing staff promotes working with a rehabilitation mindset.

Some have worked in this department for nearly a hundred years and are a fixture, but only now [after the geriatric rehabilitation education, AMV] they realize: I may not be attending the multidisciplinary team meeting […] or I am not the case manager of this rehabilitant, but I am on a nightshift and I notice that getting in and out of bed by himself is not going well yet. So I might ask: “how do you think you will manage at home?” So they have a much better understanding of all aspects of rehabilitation that are also needed at home, and they dare to take more control of it. (Participant 22)

Participants mentioned that attending training has important outcomes such as nursing staff become more aware of the quality of their work, and it contributes to the leadership of the geriatric rehabilitation nurse. Leadership is a fourth condition for the successful implementation of interventions. The participants made an appeal to all nursing staff working in geriatric rehabilitation to be aware of and show their expertise:

…what can help them take that role? I think…awareness among nurses that they are important. (Participant 4)

So you take on that role by directing and participating in the whole process. And I think if you do that, your colleagues, the other disciplines, well, you show them that: Hey, I know what I'm talking about. (Participant 5)

## Discussion

The aim of this focus group study was to explore and clarify the role of nursing staff working in geriatric rehabilitation on supporting patients in goal setting and achieving. This work was done through reflecting on interventions meant to support goal setting and achieving in geriatric rehabilitation.

### Matching Patients’ Needs

Collaboratively setting goals in the admission phase with the help of a scale (Type 1) increases patients’ awareness of why they are in this healthcare setting and the goals they could be working on. The same is true for fostering patient involvement in the rehabilitation process (Type 2). This could be supported by the use of an instrument like a short questionnaire about progression on goals. According to the participants in this study, interventions providing support for short-term goals (Type 3) have many benefits for patients. Taking into account the characteristics of the older patient, the situation (postacute), and the short time frame in which the treatment must take place given the limited reimbursement, these interventions make rehabilitation manageable for the patient and help the family to take on a supportive role. This is confirmed in various ways by other studies. Turner-Stokes et al. (2015) studied a method in which short-term goals toward the identified key goal objectives were set and reviewed at fortnightly intervals. They found that patient goal engagement improved significantly between admission and discharge. In addition, the evaluation tool for geriatric rehabilitation, developed by Janssen et al. (2019), confirms the appropriateness of working with small goals. Two of the evaluation tools’ criteria are “Rehabilitation goals are continuously coordinated with the client” and “Informal caregivers are explicitly involved in the therapy and are aware that they can practice with the client.”

### Aligning With the Role of Nursing Staff

For the nursing staff, there are three aspects of importance in applying all six interventions (Types 1, 2, and 3), and these are evident from the feedback of the participants. The first aspect is the delicate process of estimating the amount of control a patient is able to take in the goal-setting process, tuning in on that level of control and establishing conditions for the patients to take as much control as possible. Research by Thompson (2007) demonstrates that this is consistent with what patients want. Although patients prefer greater involvement in decision-making, they expect professionals to recognize that the amount of involvement varies according to the circumstances.

The second aspect that emerged from the participants is involving family in the process. Participants stressed the important role of family in setting goals as well as in working on goals. Remarkably, this aspect is only marginally described in the papers about the interventions. Dahkle et al. (2020) explored interprofessional staffs’ perceptions of interprofessional collaboration and patient-centered care and confirmed the importance of incorporating family. In both of these aspects, tuning in on the level of control a patient is able to take and involving family, the participants seemed confident and competent.

The third aspect the focus group interviews revealed that interventions that focus on advancing patient participation during the rehabilitation process (Type 2) align well with current practice. The participants expressed that they play a linking role between the patient’s personal goals and professional treatment aimed at functional recovery. This does not come as a surprise. Two of the four functions in the theoretical framework of Kirkevold (2010), which describes the nursing role in stroke rehabilitation—namely, the interpretative and the integrative function—are at their core connective in nature. Through the interpretive function, the nurse helps the patient to interpret the situation by providing them with individually adjusted information. The integrative function refers to the nurse helping the patient transfer learned techniques to daily activities (Kirkevold, 2010). The focus group interviews revealed that to optimally take on this connecting, coordinating role, interprofessional cooperation must be further developed. For example, rehabilitation plans are as yet mostly an assembling of discipline-specific goals and do not encourage nursing staff taking on a coordinating role. The current practice of working as separate disciplines is also illustrated by the participants’ ambivalence about the third type of interventions, providing support in working on short-term goals. Participants emphasized the benefits of working on short-term goals with patients but hesitate to incorporate this into their nursing practice, thus missing the opportunity to be the center of all disciplines. In the research literature, there are three factors that influence this issue and impede the nurse from taking on a coordinating role. All three factors were mentioned by the participants. First, Sinclair et al. (2009) emphasized the added value of interprofessional collaboration on patient-centered goals. This stimulates the different healthcare professionals, including the nurse, to share their knowledge and skills and thus synergistically influence the patient care provided. Second, Dahlke et al. (2020) describe that time and a perceived power imbalance between disciplines can hinder collaboration and lead to a focus on only medical issues. Implementing the investigated interventions aimed at advancing patient participation in the rehabilitation process (Type 2) and providing support for patients in working on short-term goals (Type 3) help formalize nursing tasks and responsibilities. Educating nurses in rehabilitation is an important third factor for performing the connecting role optimally. Several authors emphasize the need for specialized education (Gutenbrunner et al., 2022; Loft et al., 2018). This fosters role recognition, which, according to Doornebosch et al. (2022), is a main facilitator of interprofessional care in geriatric rehabilitation.

### Strengths and Limitations

A strength of the study is that the subject was of interest to many nursing staff working in geriatric rehabilitation. Recruiting for the focus group interviews went very smoothly. This underscores the relevance of this issue. Another strength is the use of the digital whiteboard at the start of the focus group interviews. This gave quieter participants an extra chance to voice their ideas. The study is limited because participants expressed their ideas about the interventions mainly based on their imaginative abilities. To fully understand the contribution of the nursing interventions to a more patient-centered and efficient interprofessional process, the application of these interventions should be the subject of further research.

### Implications for Practice

The results from the focus groups made clear that setting goals with the aid of a scale is more the task of the entire multidisciplinary team rather than the nursing staff. In this multidisciplinary process, specific nursing focus points are currently missing and should be incorporated into the admission process. Interventions focused on advancing patient participation (Type 2) align clearly with the coordinating role of nursing staff in the rehabilitation team. Participants emphasize the benefits of working with short-term goals (Type 3) for patients. To incorporate these into their nursing practice and strengthen the coordinating role, interprofessional teamwork needs to be further developed, available interventions should be implemented, and nursing staff should be trained in rehabilitation. Lastly, the needs and role of family in geriatric rehabilitation deserves to be embedded in interventions.

## Conclusion

The role of nursing staff in geriatric rehabilitation in the Netherlands is developing. Reflecting on the three types of interventions helped to further clarify this role. Nursing staff working in geriatric rehabilitation see themselves playing a coordinating role in the multidisciplinary team, supporting the patient in goal work. Interventions aimed at advancing patient participation (Type 2) and working toward goals (Type 3) reinforce this role.

**Key Practice Points**

- Goal setting in the admission phase is a multidisciplinary responsibility. Specific nursing focus points should be incorporated into the admission goal-setting process.
- To optimally support patients in achieving goals, interprofessional collaboration needs to be further developed.
- The needs and role of family in geriatric rehabilitation deserve to be embedded in interventions.

## Conflict of Interest

The authors declare no conflict of interest with respect to the research, authorship, or publication of this article.

**Figure:**
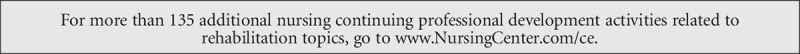


**Figure:**
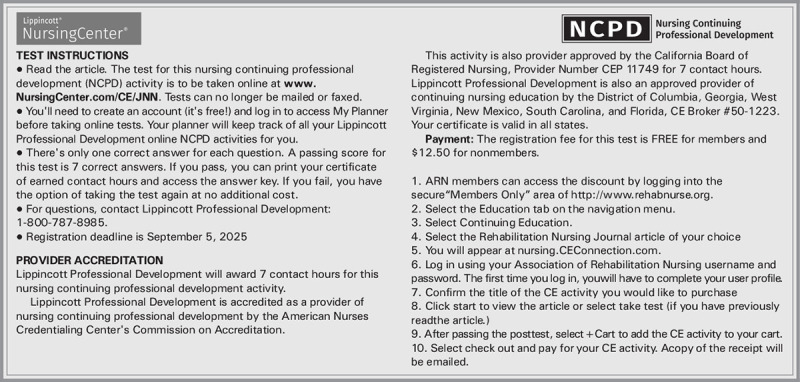


## Funding

This research was supported by ZonMW Grant 516022517, awarded to Robbert Gobbens.
